# Among the Colleges

**Published:** 1902-11

**Authors:** 


### AMONG THE COLLEGES.

KANSAS CITY VETERINARY COLLEGE is enjoying a prosperous session with an enrollment of one hundred and thirty-one students. The officers and instructors are making a special effort under these encouraging conditions to give a most thorough course of instruction. With two other colleges in Kansas City this showing of students bespeaks well of the management of this institution, the oldest in Missouri.

THE ONTARIO VETERINARY COLLEGE opened its session Wednesday, October 15, 1902, with a lecture by the Principal, Professor Andrew Smith, F.R.C.V.S. A large class of students, including representatives from various parts of North America, the Pacific coast, the West Indies, as well as from the Dominion of Canada, and some from Great Britain, listened attentively to the lecture. The session presents every indication of being a most successful one.

McGILL UNIVERSITY ANNUAL CALENDAR, thirty-seventh session —1902–03—received, in which no special changes are noted.

